# Atomization Control to Improve Soft Actuation Through Vaporization

**DOI:** 10.3389/frobt.2021.747440

**Published:** 2021-09-03

**Authors:** Han-Joo Lee, Esteban Guerra-Bravo, Arturo Baltazar, Kenneth J. Loh

**Affiliations:** ^1^ Material Science and Engineering Program, University of California San Diego, La Jolla, CA, United States; ^2^ Active, Responsive, Multifunctional, and Ordered-materials Research (ARMOR) Laboratory, University of California San Diego, La Jolla, CA, United States; ^3^ Robotics and Advanced Manufacturing Program, CINVESTAV-Saltillo, Ramos Arizpe, Mexico; ^4^ Department of Structural Engineering, University of California San Diego, La Jolla, CA, United States

**Keywords:** atomization, evaporation, modal analysis, piezoelectric, soft robotics

## Abstract

Soft actuation through droplet evaporation has significantly improved the actuation speed of methods that utilize liquid vaporization. Instead of boiling bulk liquid, this method implements atomization to disperse small droplets into a heater. Due to the large surface area of the droplets, the liquid evaporates much faster even at small temperature changes. However, further analysis is required to maximize the performance of this complex multi-physics method. This study was conducted to provide further insight into the atomizer and how it affects actuation. Numerical simulations were used to inspect the vibration modes and determine how frequency and voltage affect the atomization process. These results were used to experimentally control the atomizer, and the droplet growth on the heater surface was analyzed to study the evaporation process. A cuboid structure was inflated with the actuator to demonstrate its performance. The results show that simply maximizing the atomization rate creates large droplets on the surface of the heater, which slows down the vaporization process. Thus, an optimal atomization rate should be determined for ideal performance.

## Introduction

Soft actuation through liquid vaporization is a promising field that can replace heavy and rigid pneumatic pumps. In general, pneumatic pumps inject pressurized gas into a soft hollow structure to inflate and actuate the system (Kohll et al., 2019; Li et al., 2021; Li et al., 2018; Mosadegh et al., 2014; Polygerinos et al., 2017; Tolley et al., 2014; Wehner et al., 2014). Although fast and accurate, actuation through pneumatic pumps requires the structure to be tethered to a pump, which limits its application. As an alternative, actuation by liquid vaporization is composed of a hollow structure that is partially filled with liquid with a low boiling point (Boyvat et al., 2019; Cartolano et al., 2019; Han et al., 2019; Nishikawa and Matsumoto, 2019; Chellattoan et al., 2020; Li et al., 2020; Mirvakili et al., 2020; Narumi et al., 2020; Mirvakili et al., 2021). Simply heating the system will vaporize the liquid and inflate the system. Depending on the application, the system can be actively heated with an embedded heater, or it can be heated by an external source. The heat propagates from the heat source and starts to increase the temperature of the liquid upon powering the system. Although a small amount of evaporation occurs from the liquid surface during small temperature change, the liquid is generally heated until it boils to speed up the vaporization. The essential components, such as the battery and microcontroller, can be easily packaged into a portable device versus a pneumatic pump that is typically bulkier.

Despite these benefits, there are several limitations that prevent the actuation method from practical applications. First, the actuation speed is considerably slower as compared to other methods, such as pneumatic pumps. This is due to the significant delay between powering the heater and heating the liquid to boiling. In addition, heating can be delayed further when considerable heat loss is present due to the environment (*e.g.*, cold climate, windy environment, or underwater). Second, the permeability of the soft elastomers drastically shortens the lifespan of the system. Over time, the embedded liquid and vapor diffuses through the material, resulting in an empty chamber without liquid. In case of large chambers, the liquid can be recharged by injecting more liquid, removing the same volume of gas, and sealing the hole created by the syringe. Last, the reversing process takes a long time, since the vapor has to condense back into liquid. In general, the system is turned off, and air-cooling slowly returns it back to its original shape.

Nevertheless, several studies introduced unique methods to improve actuation through liquid vaporization. In an earlier study (Lee and Loh, 2021), implemented a thermoelectric device to heat, as well as actively cool down the system. The thermoelectric device was installed on the bottom of the system to control heat flow, whereas the other layers consisted of a double-walled elastomer, preventing undesired heat loss during actuation. This method successfully actuated a structure underwater and shortened the cooling process by ∼62%. Another study by (Lee et al., 2020) implemented droplet evaporation instead of boiling the entire liquid. Vibrating mesh atomization was used to disperse the liquid into small droplets that were ejected into a heater. The cross-section of the heater was designed to maximize the evaporation process of the droplets. Due to the large surface area of the droplets, rapid actuation was achieved even at much lower temperatures. This method showed immense potential by performing similar to conventional pneumatic pumps.

This study aims to provide further insight on the previous study that used mesh atomization and determine the optimal atomization rate for soft material actuation. While there are several approaches to optimize this multi-physics process, this work focuses on how the atomization rate affects droplet evaporation on the heater surface. First, a finite element method (FEM) was used to study the vibration modes of the atomizer. These results were compared with experimentally measured atomization rates for validation. The results obtained from the numerical simulation was used to control the atomizer, and the collected droplets on the heater surface were analyzed through an optical microscope. Next, the system was installed inside a cuboid system, and the actuation was compared to study the effect of droplet growth and evaporation.

## Materials and Methods

### Experimental Setup

**FIGURE 1:**
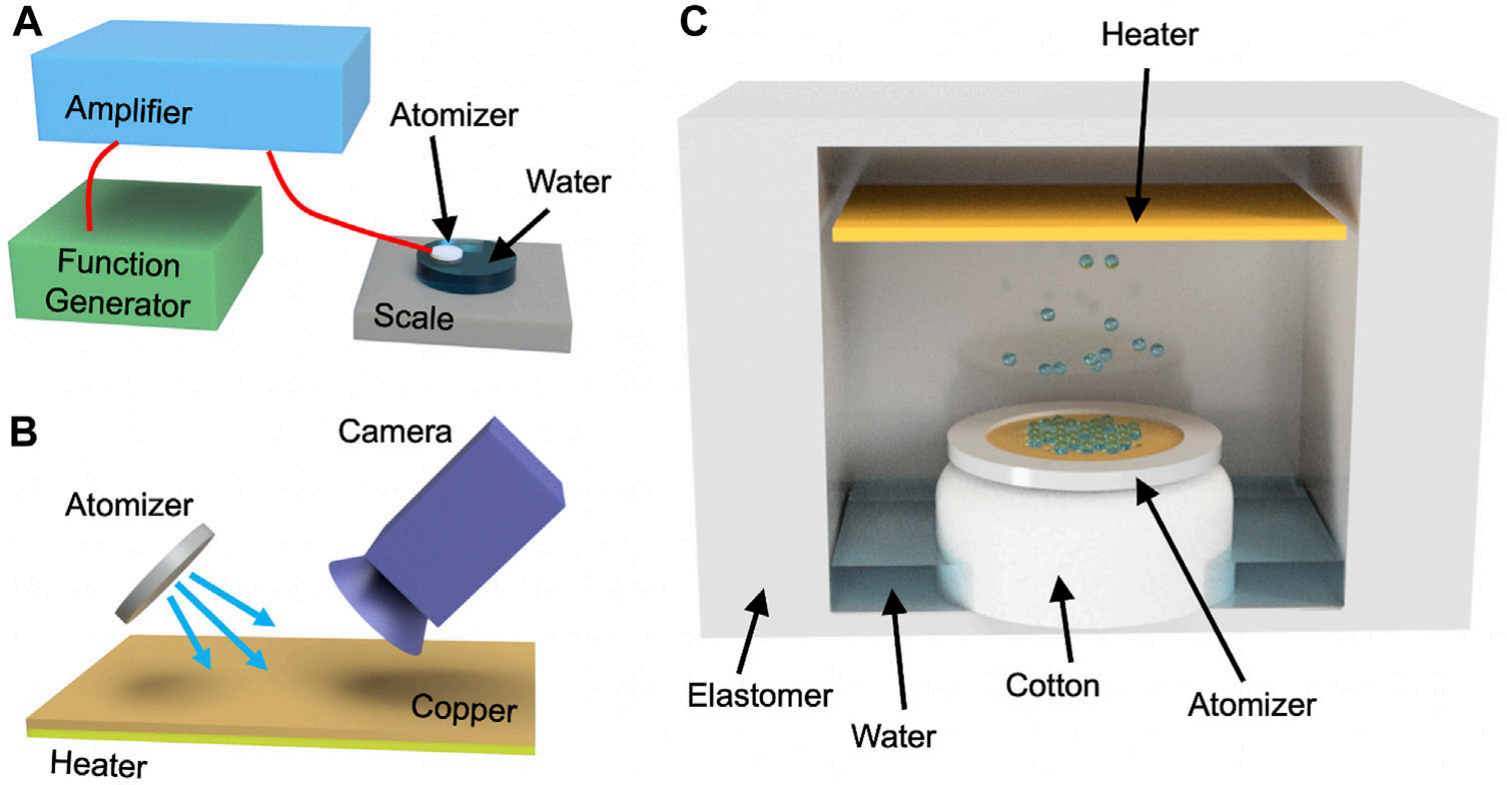
**(A)** The schematic shows the setup for measuring the atomization rate. A function generator was connected to an amplifier, which powered the atomizer. **(B)** The size of the droplets on the heater surface was measured with an optical microscope. The droplets were sprayed at an angle for the camera to record. **(C)** The image shows the cross-section of the actuator. The atomizer was fixed in place above the cotton wick, which absorbed the embedded water. The droplets were ejected to the heater, and the vapor inflated the structure.

A commercial vibrating mesh atomizer was used in this study. Compared to ultrasonic atomization, mesh atomization is used in many applications due to their lower power consumption. The most popular applications include humidifiers (Zhang et al., 2013) and nebulizers (Le Brun et al., 2000). The mesh atomizer used in this study consisted of a piezoelectric ring and a metal mesh at the center. Similar to most vibrating mesh atomizers, the mesh consisted of conical holes that had a diameter of ∼80 μm at the bottom and ∼10 μm at the top. The atomizer is normally positioned so that the bottom layer of the mesh is in contact with liquid. When the ring starts to vibrate, small droplets are ejected into the air through the holes. The setup to measure the atomization rate is shown in Figure 1A. The alternating current (AC) voltage that controls the mesh atomizer was produced by a Keysight 33210A function generator, which was amplified with an Electronics and Innovation 500S06 amplifier to power the piezoelectric ring. The atomizer was placed on the surface of the water inside a plastic petri dish. This petri dish was placed above a Mettler Toledo ME204E scale to measure weight loss. The results were plotted over time, and the linear slope was measured to quantify the atomization rate.

In order to study the evaporation process, the sizes of the droplets were measured at the heater surface, and the setup is shown in Figure 1B. The heater consisted of nichrome wires that generated heat when current was applied. In this work, 3 V was applied to the heater using an Agilent E3642A power supply, which was equivalent to ∼0.34 A of current. A strip of 3M copper tape of high thermal conductivity was applied to the surface of the heater to better visualize the droplets. The atomizer was positioned so that it sprayed droplets to the heater at an angle. The droplets were collected at the heater surface, and their size was measured with an optical microscope. The sizes of the droplets were measured by calculating the average of the longest diameter of ∼50 droplets. The temperature change at the surface was also measured with an Omega thermocouple throughout the test.

Actuation performance was measured by sealing the atomizer and heater inside a soft silicone elastomer, which was similar to a previous study (Lee et al., 2020). The setup is shown in Figure 1C, where the atomizer was fixed in place above a cotton wick. After sealing the structure, 1 ml of water was injected into the chamber. The wick absorbed the embedded water so that the mesh can atomize the liquid. A flat compact heater was installed above the atomizer to evaporate the droplets. Although heater designs with complex cross-sections are expected to improve the evaporation process, a simple flat heater was used in this study to simplify the conditions. These components were sealed with Dragon Skin FX-Pro to achieve inflation during vaporization. The uncured elastomer was poured into 3D-printed molds to form the shapes. The side walls were ∼6 mm thick, whereas the top layer was ∼0.5 mm thick. The difference in thickness concentrated the deformation to the top layer, which was measured during testing. A video was recorded throughout the test, and image processing was used to calculate the displacement.

### Finite Element Model

**FIGURE 2:**
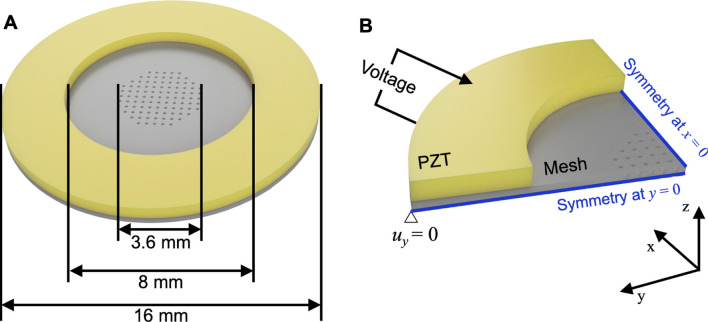
**(A)** The schematic shows the dimension of the atomizer. **(B)** The image shows how the atomizer was modeled in *ANSYS*. The voltage was applied to the top and bottom layers of the piezoelectric ring.

The detailed dimension of the atomizer is shown in Figure 2A. The piezoelectric ring had an outer diameter of 16 mm and an inner diameter of 8 mm. The thickness of the ring was 0.63 mm, and the thickness of the metal mesh was 50 µm. The holes of the mesh were within 1.8 mm from the center of the disc. The mesh had 551 number of conical holes with ∼80 μm on the bottom and ∼10 μm on the top. Figure 2B shows the FEM in *ANSYS*. A quarter of the atomizer was modeled with *x* and *y*-axis symmetry. The displacement of the bottom edge was fixed in the *y*-direction, and voltage was applied to the top and bottom layers of the piezoelectric ring. The quarter of the piezoelectric ring was mapped using 675 number of SOLID5 hexahedral elements of eight nodes per element, and the stainless-steel disc consisted of 70,341 number of SOLID186 tetrahedral elements of eight nodes per element with quadratic displacement.

The linear constitutive relations that define the coupling between mechanical stress, mechanical strain, electric field, and electric displacement are given as (Rupitsch, 2019):

$$\sigma =\;\left[C^{E}\right]\cdot \varepsilon -\left[e\right]\cdot E$$ (1)

$$D={\left[e\right]}^{T}\cdot \varepsilon +\left[\xi^{S}\right]\cdot E$$ (2)

where ***σ*** is stress tensor, ***D*** is electric displacement vector, ***ε*** is strain tensor, ***E*** is electric field, [*C* ^*E*^] is elastic constant at constant electric field, [*e*] is piezoelectric stress coefficients, *T* is transpose of *e*, and [*ξ* ^*S*^] is dielectric tensor at constant mechanic strain. The mechanical and electrical balance of the system can be defined using the following equations:

$$\rho \ddot{u}=\nabla \cdot \sigma$$ (3)

∇⋅D=0 (4)

where *ρ* is density, and $\ddot{u}$ is vector of acceloration. In the stationary harmonic analysis, cyclic loading was applied to the structure. The complex responses *x*_*1*_ and *x*_*2*_ are solved from the following matrix equation.

$$\left(-{\text{Ω}}^{2}\left[M\right]+j\text{Ω}\left[\text{C}\right]+\left[\text{K}\right]\right)\left\{x_{1}+jx_{2}\right\}=\left\{F_{1}+jF_{2}\right\}$$ (5)

where [*M*] is mass matrix, [*C*] is damping matrix, [*K*] is stiffness matrix. The load (*F*_*1*_ *+jF*_*2*_) is applied by the sinusoidal voltage with a freqency of *Ω* A frequency sweep of voltage was applied to the piezoelectric ring to analyze the vibration modes. The contact between the piezoelectric ring and metal disc was perfectly bonded. The displacement at the center of the metal mesh was recorded to visualize the results. Vibration modes of frequencies showing displacement peaks were also obtained. The parameters for the piezoelectric ring and stainless steel were obtained from parameters reported in the literature (Li and Li, 2015; Dou et al., 2019). A density of 7,980 kg/m^3^, Young’s modulus of 186.8 GPa, and Poisson’s ratio of 0.31 were used for the stainless steel. A density of 7,500 kg/m^3^ and Poisson’s ratio of 0.32 were used for the piezoelectric ring. Detailed information on dielectric constant, piezoelectric constant, and elastic constant can be found in (Li and Li, 2015).

## Results and Discussion

### Vibration Modes

**FIGURE 3:**
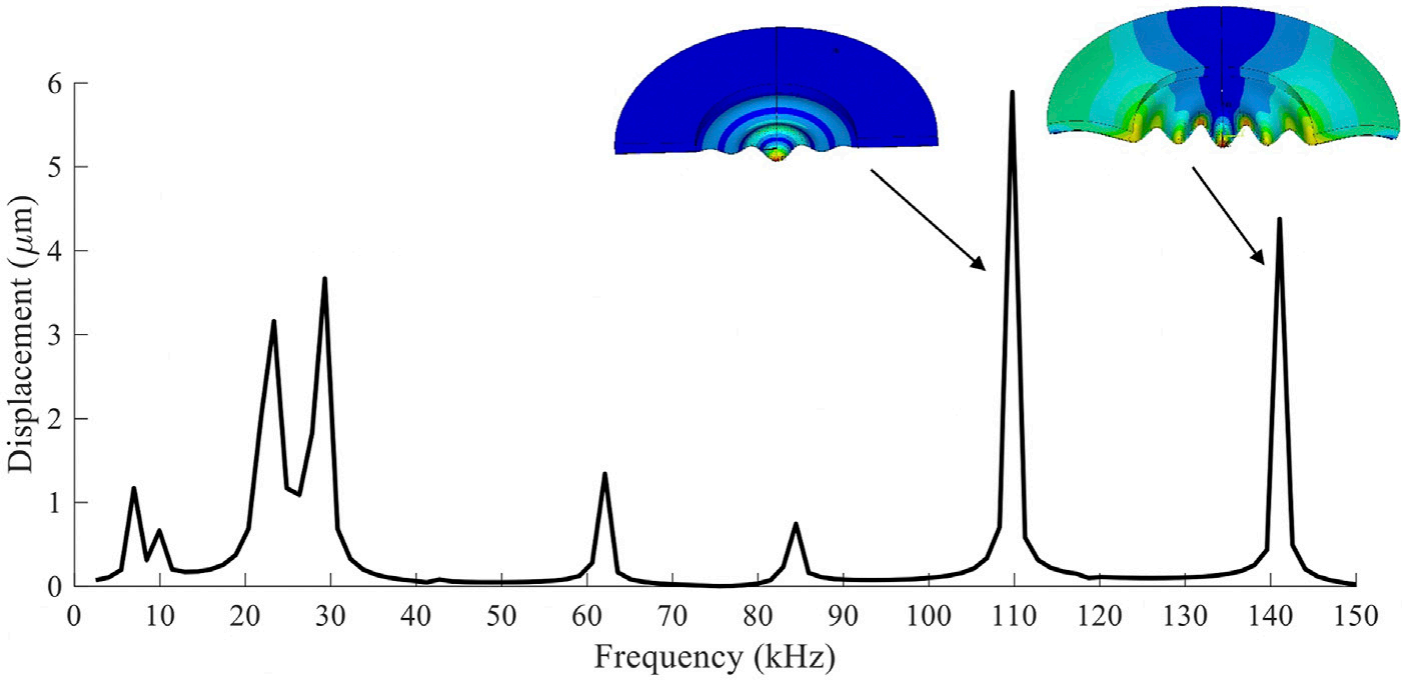
The displacement at the center of the mesh was measured through FEM during a frequency sweep. The vibration modes of the two largest peaks are also shown.

Figure 3 shows the FEM result of a wide frequency sweep when 20 V of AC voltage was applied to the PZT ring. The displacement at the center of the disc was measured to represent the modes. The result shows several resonant frequencies from 0 to 150 kHz, where 110 and 141 kHz showed the highest peaks. The max displacement at 110 kHz reached ∼6 μm, while 141 kHz reached ∼4.2 μm. The vibration modes of the two peaks are also shown in Figure 3. At 110 kHz, the displacement was focused near the center of the disc. On the other hand, displacement at 141 kHz was relatively distributed evenly throughout the mesh. In addition, the piezoelectric ring also showed a small amount of displacement. The shapes of the cross-section are also expected to affect the atomization rate, since holes are concentrated near the center of the disc. Higher displacement at the center will facilitate higher atomization rate (Yan et al., 2019). The next two highest peaks were at much lower frequencies between 20 and 30 kHz. However, the actual experiment did not show any atomization at these frequencies. Therefore, the experiments performed hereafter were focused on the frequency range between 100 and 150 kHz.

### Displacement and Atomization Rate

**FIGURE 4:**
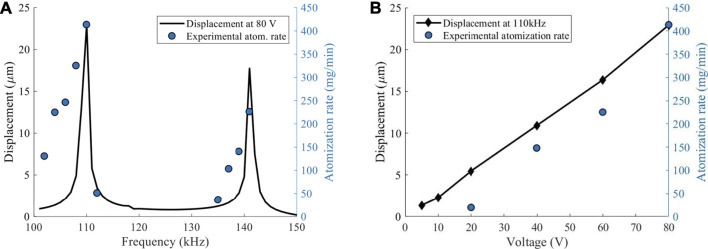
**(A)** The displacement of the center of the disc obtained from FEM is plotted together with the experimentally measured atomization rate. A voltage of 80 V was used for both results. **(B)** The plot shows displacement at the center of the disc at different frequencies. The results are compared with the atomization rate.

The atomization rate was quantified by measuring weight loss during atomization, and the results are shown in Figure 4A. The atomization rate was measured at 2 kHz intervals from 100 to 150 kHz. The voltage applied to the atomizer was 80 V. Any frequencies without data points did not show any visible atomization. For comparison, an FEM result similar to Figure 3 is also shown when 80 V was applied to the piezoelectric ring. The highest displacements at 110 and 140 kHz were ∼23 and ∼18 μm, respectively. Similar to the FEM result, the atomization rate demonstrated two peaks at 110 and 141 kHz. The atomization rate at 110 kHz was ∼400 mg/min, whereas at 141 kHz, it was ∼250 mg/min. Although the frequencies of the maximum peaks were in good agreement with the FEM results, the shape of the spectra distribution showed some discrepancies. These small differences were expected as the actual atomizer used epoxy to bond the piezoelectric ring to the metal mesh. In addition, the wires that were soldered to the ring also affected its vibration behavior.

The effect of voltage was studied at a frequency of 110 kHz, since it showed the largest amount of displacement and atomization rate. The displacement at voltages of 5, 10, 20, 40, 60, and 80 V were obtained through FEM. The trend was fairly linear, and the displacement at 80 V was the same as the result in Figure 4B. The atomization rate also decreased in a linear trend with decreasing voltage. However, the linear fit of the experimental result showed that a minimum of ∼18 V was required for atomization. The results from Figure 4 show that the FEM can be used to determine the optimal frequency of the atomizer, while increasing the driving voltage will linearly increase atomization rate.

### Droplet Analysis

**FIGURE 5:**
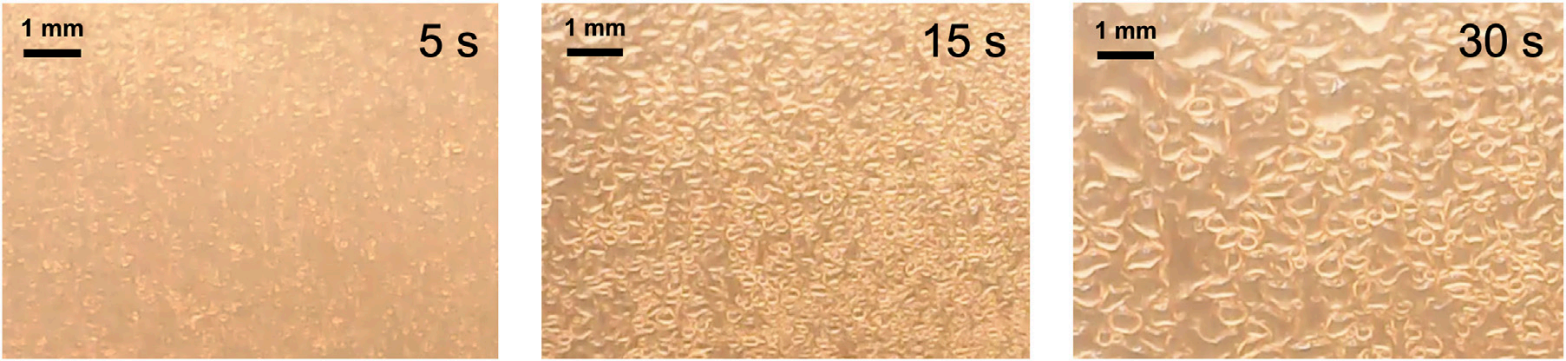
The images show the droplets at the heater surface when the atomizer was powered with 80 V. Power of ∼1 W was applied to the heater. The droplets quickly started to grow over time.

**FIGURE 6:**
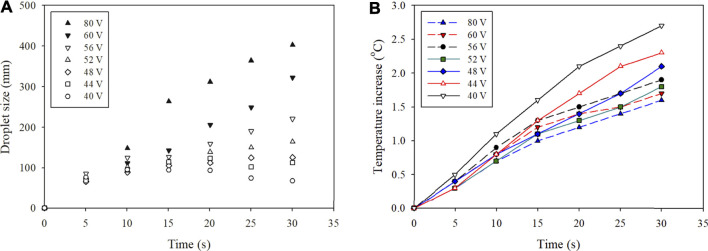
**(A)** The sizes of the droplets were measured over time. The voltage that powered the atomizer varied from 40 to 80 V. **(B)** The temperature of the heater was also measured throughout the test.

The droplet size on the heater surface was studied in this section to analyze the effect of atomization rate on evaporation. The voltage applied to the atomizer varied from 40 to 80 V, and it was powered simultaneously with the heater. Figure 5 shows images of the droplets when atomizing water using 80 V. At this highest voltage, the growth of droplets was higher than the evaporation rate. As a result, the size of the droplets continued to grow over time. The size started at ∼70 μm at 5 s and quickly grew to ∼400 μm after 30 s. Table 1 shows the average and standard deviation of the droplet sizes measured throughout the test. The results are better visualized in Figure 6A. At 40 V, the size of the droplets initially started to grow but decreased after ∼10 s. The droplet size at this lowest voltage remained less than 100 μm throughout the test. At 44 V, the size tended to plateau after ∼20 s, and the size increased continuously at 48 V or higher.

**TABLE 1:** Droplet size on the heater surface was measured over time during atomization.

Time Voltage	5 s		10 s		15 s		20 s		25 s		30 s	
	Aver. (μm)	Std. (μm)	Aver.	Std.	Aver.	Std.	Aver.	Std.	Aver.	Std.	Aver.	Std.
40 V	77.1	14.4	95.7	21.8	94.8	26.0	93.9	23.0	74.5	20.3	67.7	13.4
44 V	67.7	13.4	94.8	18.2	114.7	29.8	123.3	36.7	102.0	15.1	113.0	19.7
48 V	66.5	15.0	89.0	21.3	105.2	18.2	112.7	26.6	124.9	19.5	126.1	41.4
52 V	76.0	14.0	102.8	15.9	114.4	32.0	139.0	44.4	150.3	41.1	164.7	43.2
56 V	86.2	20.5	124.9	32.6	127.1	44.9	159.4	54.9	190.3	91.1	220.8	50.3
60 V	71.1	13.0	111.6	38.3	142.9	51.3	205.9	91.8	248.9	84.6	322.3	111.3
80 V	73.8	15.6	147.9	58.1	263.8	100.7	311.7	119.5	364.1	104.4	402.4	117.9

The temperature of the heater was also measured during the tests, and the results are shown in Figure 6B. The test conducted with 40 V showed the largest temperature increase over time and eventually reached ∼2.7°C. Considering that the boiling temperature of water is 100°C, this very small increase in temperature was enough to evaporate the small droplets. The lowest atomization rate showed the slowest growth rate of the droplets, and the size started to decrease as temperature continued to rise. Experiments at higher atomization rates showed different trends when the droplet growth rate was larger than the evaporation rate. The surface area of the droplets starts to decrease with increasing droplet size, which significantly slows down the evaporation rate at the liquid surface. As a result of heating large droplets, the highest atomization rate at 80 V showed the lowest temperature increase after 30 s. These results show that evaporation rate converges to a maximum at a certain atomization rate. When higher voltage is applied to the heater, the optimal voltage to power the atomizer is expected to increase. Although this test was conducted in an open system, the buildup of droplets on the surface of the heater is expected to be present even in a closed system.

### Soft Actuation Through Evaporation

**FIGURE 7:**
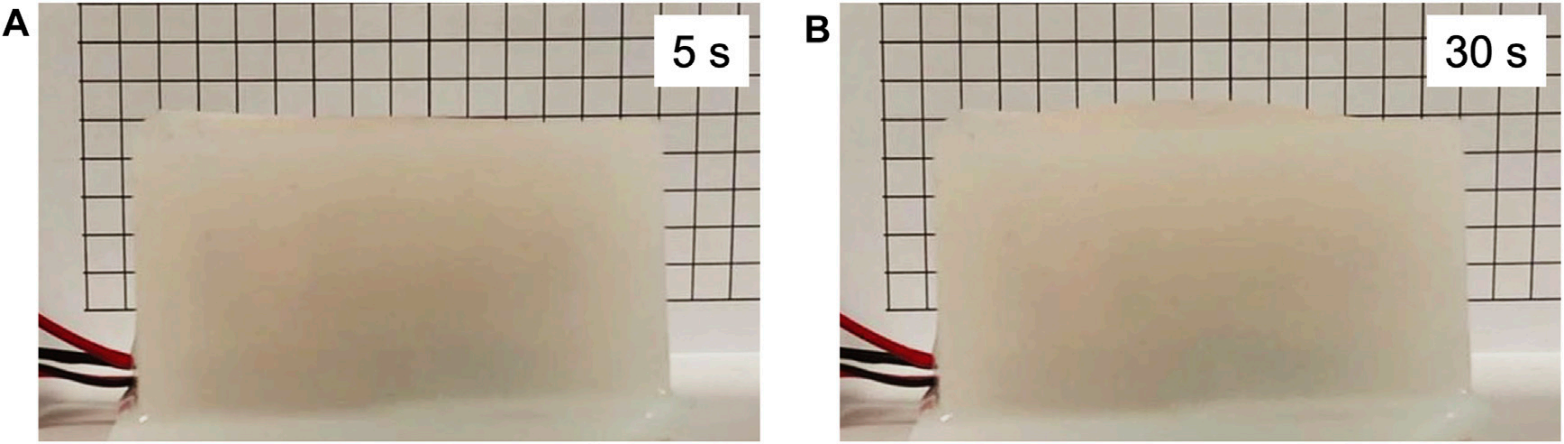
**(A)** The fabricated structure is placed in front of a grid with lines separated by 5 mm. **(B)** The image shows the inflated structure after 30 s of actuation.

Figure 7A shows the fabricated structure placed in front of a grid with grid spacings of 5 mm. Voltages from 70 to 90 V were applied to the atomizer at 2 V intervals, and 3 V applied to the heater resulted in 0.6 A of current. The structure after 30 s of actuation is shown in Figure 7B. Similar to the droplet size test in the previous section, the heater and atomizer were powered simultaneously. The deformation of the cuboid structure was concentrated on the top layer due to its thinness. Inflation was recorded with a video camera, and image processing was used to determine the maximum displacement at the center. Since the method used water instead of ethanol, which was used in a previous study by (Lee et al., 2020), the rate of inflation was visibly slower. Nevertheless, the measured displacements provide information on how atomization rate affects actuation.

**FIGURE 8:**
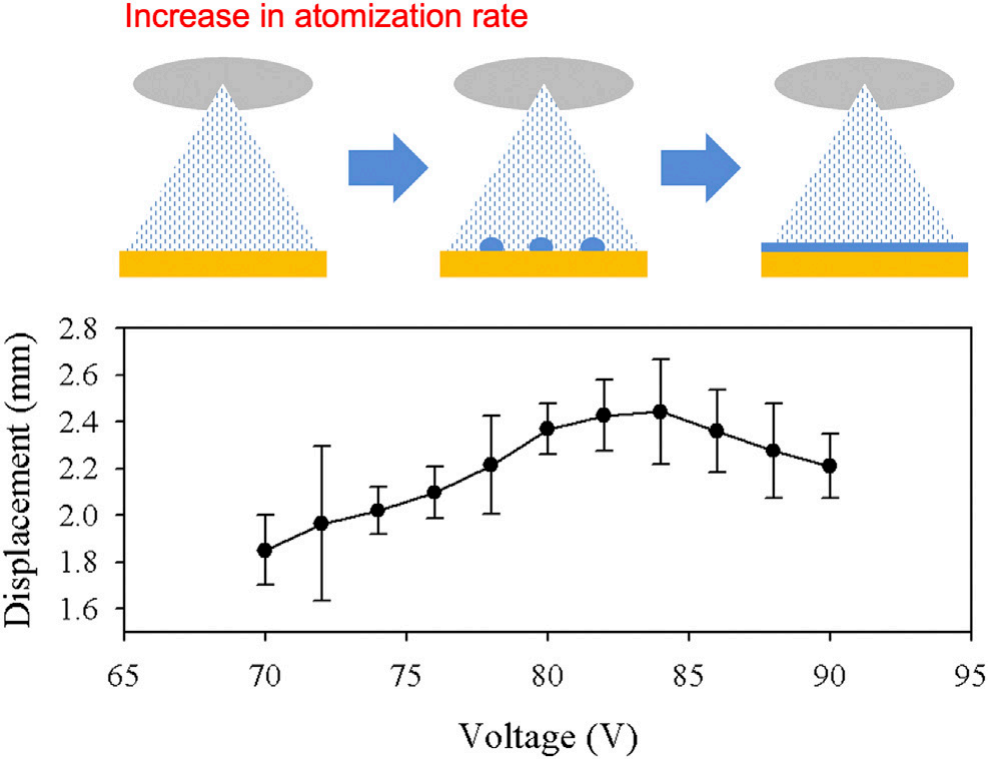
The displacement of the top layer after 30 s of actuation is shown in the plot. Higher voltage applied to the atomizer resulted in higher atomization rate. The large growth of the droplets at the heater surface slowed down the actuation speed.

In order to represent the actuation performance, the displacement of the top layer after 30 s of actuation is plotted in Figure 8. Each condition was tested five times to calculate the average and error. It was expected that the compact heater with higher power consumption used in the actuator reached higher temperatures in the closed system. The displacement increased linearly up to 84 V, which showed a maximum displacement of ∼2.44 mm. The displacement started to decrease beyond this point, which reached ∼2.21 mm at 90 V. The illustration above the plot represents the condition explained in the previous section. At lower atomization rates, the growth of droplets on the heater surface was lower than the evaporation rate. However, these droplets are expected to grow larger after a certain threshold. Continued increase in atomization rate will form larger droplets at the heater surface, which decreases the evaporation rate due to lower surface area. Eventually, evaporation is expected to significantly decrease when a thin liquid layer is formed on the heater surface, preventing the atomized droplets to come in contact with the heater. In the system presented in this work, ∼84 V appeared to be the optimal voltage that maximized the evaporation process.

## Conclusion

This work aims to address and analyze the importance of atomization rate in a novel soft actuation method that utilizes droplet evaporation to drastically enhance actuation through liquid vaporization. While the method showed promising results to significantly speed up the vaporization process, further optimization was required to take full advantage of rapid vaporization. In this study, the effect of atomization rate on droplet growth at the heater surface was studied to maximize the performance of the system. First, FEM was used to analyze the vibration modes of the atomizer. The simulation successfully predicted the resonant frequency of the system, which was chosen as the driving frequency for the piezoelectric ring. Increasing the voltage in the FEM showed a linear increase in displacement at the center of the disc. Although the experimental atomization rate showed a threshold, it also demonstrated a linear increase with increasing voltage. Second, the droplet growth was measured on the heater surface during atomization. At lower voltages, the size of the droplets eventually started to decrease over time. This is due to the constant heat generated by the heater, which steadily increased temperature. When the atomizer was powered with higher voltages, however, the droplets steadily started to grow on the heater surface over time. These results show that simply maximizing the atomization rate will impede the evaporation process. This effect was demonstrated by measuring the displacement of a soft actuator. The displacement of the system increased with increasing atomization rate until it reached a maximum value. Powering the atomizer with voltages beyond this point resulted in lower actuation rates. Therefore, the optimal atomization rate should be first determined for a given heating condition to maximize the performance of the actuator. With further studies, the method is expected to show rapid actuation even with water, which has higher boiling temperature but is safe and easy to access.

## Data Availability

The raw data supporting the conclusions of this article will be made available by the authors, without undue reservation.
